# Differentiation, Distribution and γδ T Cell-Driven Regulation of IL-22-Producing T Cells in Tuberculosis

**DOI:** 10.1371/journal.ppat.1000789

**Published:** 2010-02-26

**Authors:** Shuyu Yao, Dan Huang, Crystal Y. Chen, Lisa Halliday, Gucheng Zeng, Richard C. Wang, Zheng W. Chen

**Affiliations:** 1 Department of Microbiology and Immunology, Center for Primate Biomedical Research, University of Illinois College of Medicine, Chicago, Illinois, United States of America; 2 Biologic Resources Laboratory, University of Illinois, Chicago, Illinois, United States of America; Johns Hopkins School of Medicine, United States of America

## Abstract

Differentiation, distribution and immune regulation of human IL-22-producing T cells in infections remain unknown. Here, we demonstrated in a nonhuman primate model that *M. tuberculosis* infection resulted in apparent increases in numbers of T cells capable of producing IL-22 *de novo* without *in vitro* Ag stimulation, and drove distribution of these cells more dramatically in lungs than in blood and lymphoid tissues. Consistently, IL-22-producing T cells were visualized *in situ* in lung tuberculosis (TB) granulomas by confocal microscopy and immunohistochemistry, indicating that mature IL-22-producing T cells were present in TB granuloma. Surprisingly, phosphoantigen HMBPP activation of Vγ2Vδ2 T cells down-regulated the capability of T cells to produce IL-22 *de novo* in lymphocytes from blood, lung/BAL fluid, spleen and lymph node. Up-regulation of IFNγ-producing Vγ2Vδ2 T effector cells after HMBPP stimulation coincided with the down-regulated capacity of these T cells to produce IL-22 *de novo*. Importantly, anti-IFNγ neutralizing Ab treatment reversed the HMBPP-mediated down-regulation effect on IL-22-producing T cells, suggesting that Vγ2Vδ2 T-cell-driven IFNγ-networking function was the mechanism underlying the HMBPP-mediated down-regulation of the capability of T cells to produce IL-22. These novel findings raise the possibility to ultimately investigate the function of IL-22 producing T cells and to target Vγ2Vδ2 T cells for balancing potentially hyper-activating IL-22-producing T cells in severe TB.

## Author Summary

TB remains one of the leading causes of morbidity and mortality among infectious diseases worldwide. Protective immunity against TB in humans remains poorly characterized, although the importance of CD4 T cells is well described in HIV-related TB. Similarly, little is known about the role of newly-defined T helper 17 (Th17) cells producing pro-inflammatory cytokine IL-17 or IL-22 during *M. tuberculosis* infection of humans. Understanding immune response, kinetics, distribution and regulation of Th17 cells will help to elucidate immune function of these cells in TB, and to explore Th17-targeted immunotherapeutics. We therefore employed the nonhuman primate TB model to study Th17 cells and their interaction with Vγ2Vδ2 T cells, a major human γδ T-cell subset having regulatory potential. We demonstrated that *M. tuberculosis* infection drove induction and distribution of IL-22-producing T cells in blood, airway, lung, lymph node and spleen. IL-22-producing T cells were most frequent in lungs, and involved in TB granuloma formation. We further showed that activation of Vγ2Vδ2 T cells could remarkably down-regulate IL-22-producing T cells in blood and tissue lymphocytes, and that such a down-regulation was mediated by an IFNγ-associated cytokine network. Our findings provide new information regarding kinetics, distribution and immune regulation of IL-22-producing T cells in TB.

## Introduction

IL-22 is a member of IL-10 cytokine family and primarily produced by Th17 T cells [1]. IL-22 signals though its heterodimer receptor comprised of IL-22R1 and IL-10R2 [2], with the IL-22 binding effect dictated by IL-22R1 that are expressed mainly on skin and mucosal epithelial cells such as digestive system, respiratory system and kidney but not on immune cells [1],[3],[4]. Upon binding to its receptor, IL-22 exerts its effect by activating STAT signal transduction pathways [2],[4]. Accumulating evidence suggests that IL-22 can be either pathogenic/inflammatory or protective depending upon environmental and host conditions. IL-22 has the ability to induce antimicrobial peptide β-defensin 2 and 3 or psoriasin in keratinocytes [2],[4],[5], and up-regulate host defense genes such as Lcn2 (encoding lipocalin-2) [6],[7]. IL-22 can also induce expression of acute phase reactants to protect against acute liver inflammation [2],[3],[4]. On the other hand, IL-22 can induce inflammatory effects. IL-22 is indeed a major inflammatory mediator in dermal inflammation and acanthosis in mouse models [8],[9]. However, it is not clear how IL-22-producing T cells involve or orchestrate host immune response. Since the subset of murine T cells producing IL-22 also produce IL-17 [1], knowledge about the IL-22-producing T cells is mainly derived from studies of IL-17-producing Th17 cells in autoimmune and inflammatory diseases. In fact, development, function and immune regulation of human IL-22-producing T cells in infections remain largely unknown.

Tuberculosis remains one of the leading causes of morbidity and mortality due to infectious diseases, with 8 million new cases and >2 million deaths reported word-wide each year [10],[11]. Although human CD4 T cells have been shown to be important for protection against adult form of pulmonary tuberculosis [12], the role of IL-22-producing T cells in TB is not known. While IL-17 and IL-23 were investigated in mouse TB model [10],[13], we have recently demonstrated that severe tuberculosis induces unbalanced up-regulation of immune gene networks and over-expression of IL-22 in nonhuman primates [14]. It has also been reported that Th17 cells producing IL-22 and IL-17 can be detected by *in vitro* antigen re-stimulation-based intracellular cytokine staining (ICS) in BCG-vaccinated and *M. tuberculosis*-infected humans [15]. However, differentiation kinetics, local/systemic distribution, and immune regulation of human IL-22-producing T cells during *M. tuberculosis* infection remain unknown. Elucidation of these aspects may potentially device immune regulatory strategy in which immune responses of IL-22-producing T cells can be balanced to facilitate protective response but minimize inflammatory consequence in tuberculosis.

Vγ2Vδ2 T cells exist only in primates and constitute 60–95% of circulating human γδ T cells [16],[17]. Studies from us and others suggest that Vγ2Vδ2 T cells play a role in mediating anti-microbial immune responses [18],[19],[20],[21],[22],[23]. Vγ2Vδ2 T cells can be specifically activated by certain low m.w. foreign- and self-nonpeptidic phosphorylated metabolites of isoprenoid biosynthesis [e.g. (*E*)-4-hydroxy-3-methyl-but-2-enyl pyrophosphate (HMBPP) and isopentenyl pyrophosphate (IPP)] [24],[25],[26],[27]. We have shown that HMBPP produced by *M. tuberculosis* and other microbes is associated with antigen presenting cell (APC) membrane and recognized by Vγ2Vδ2 TCR [28]. We have also demonstrated that HMBPP-specific Vγ2Vδ2 T cells can readily migrate and accumulate in the pulmonary compartment during *M. tuberculosis* infection, and that rapid recall expansion of these cells is associated with immunity against fatal tuberculosis in juvenile rhesus monkeys [21],[29]. More recently, we reported that HMBPP activation of Vγ2Vδ2 T cells can antagonize IL-2-induced CD4+CD25+Foxp3+ T regulatory cells in mycobacterial infection [30], suggesting that Vγ2Vδ2 T cells may play a regulatory role as well in immune responses against tuberculosis. Our findings in the macaque TB model system raise the possibility to study cell-cell interaction and mutual regulation between Vγ2Vδ2 T cells and IL-22-producing T cells during *M. tuberculosis* infection.

In the current study, we first demonstrated differentiation kinetics and local/systemic distribution of IL-22-producing T cells during *M. tuberculosis* infection of macaques. We then provided the first evidence indicating that effector function of IL-22-producing T cells differentiated from *M. tuberculosis* infection was susceptible to down-regulation by HMBPP activation of Vγ2Vδ2 T cells. We also elucidated an immune mechanism underlying HMBPP/Vγ2Vδ2 T-cell-mediated immune regulation of IL-22-producing T cells.

## Results

### *M. tuberculosis* infection drove apparent increases in numbers of T cells capable of producing IL-22 or IL-17 *de novo* without *in vitro* Ag stimulation in the blood and BAL fluid

**Figure 1:**
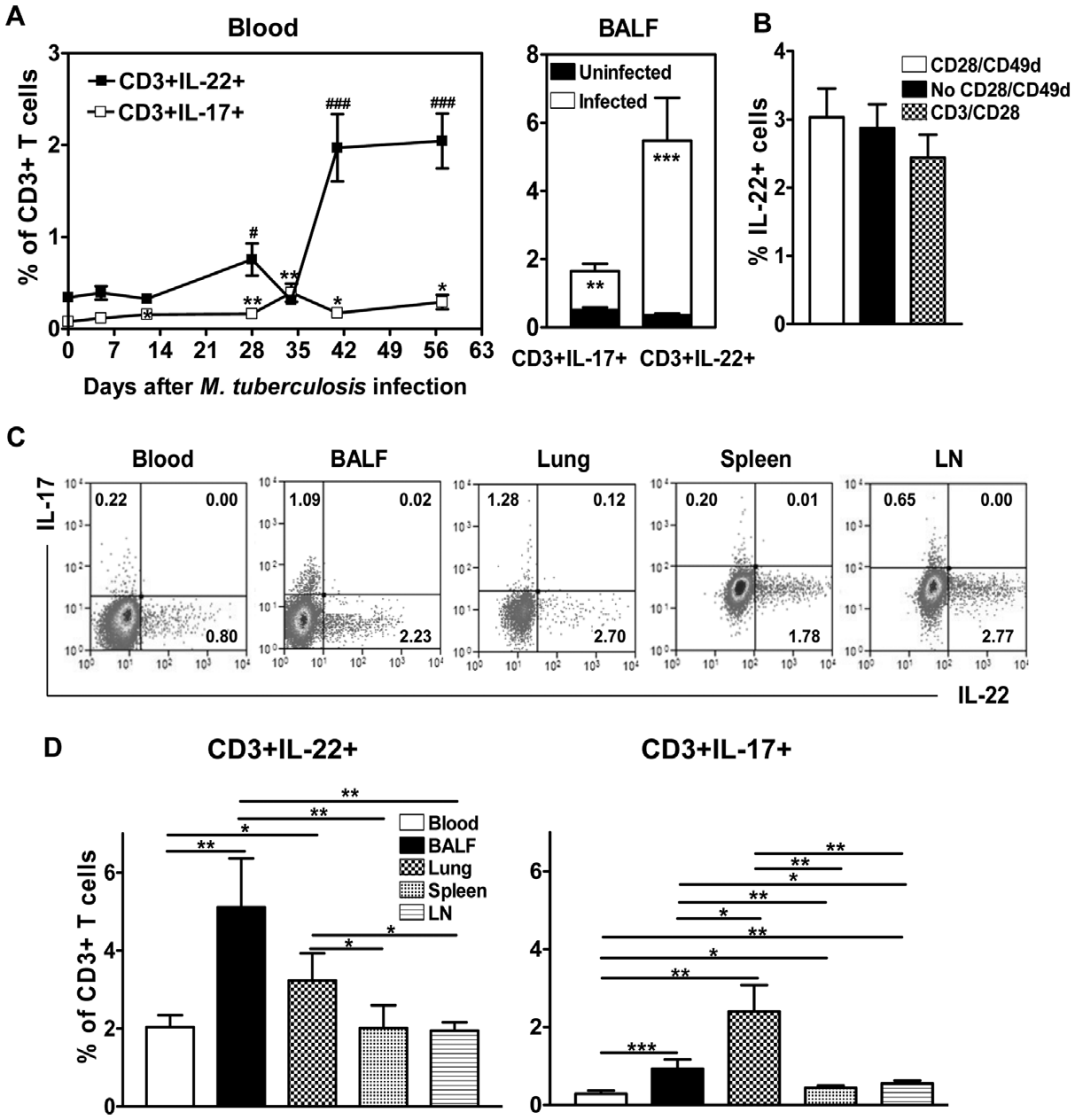
*M. tuberculosis* infection resulted in apparent increases in numbers of T cells capable of producing IL-22 without *in vitro* Ag re-stimulation, and drove distribution of these cells more dramatically in lungs than in blood and lymphoid tissues. (**A**) Graphic data show percentage numbers of T cells capable of producing IL-22 or IL-17 *de novo* in the blood (left) and BAL fluid (right) during *M. tuberculosis* infection. The cells were directly measured by ICS in the presence of CD28/CD49d but without *in vitro* Ag stimulation. This would allow direct evaluation of regulatory effect by Vγ2Vδ2 T effector cells in the presence or absence of HMBPP. Data are mean percentage numbers in CD3 T cells with error bars of SEM derived from nine cynomolgus macaques. Data were gated on CD3 although most IL-22 cells were CD4 T cells (Fig. S1). This would allow direct comparisons with Vγ2Vδ2 T cells within CD3 T-cell population as shown in Figs. 3–5. CD3+ T cells producing IL-22 or IL-17 in this and other figures included Vγ2Vδ2 T cells. The magnitude of increase in IL-22+ T cells in PBL (^#^) at weeks 4, 6 and 8 and in BAL fluid at week 8 were significantly greater than that in IL-17+ T cells (*) (P<0.005). ^#^ or *, P<0.05, **, P<0.01, ^###^ or ***, P<0.001. (**B**) Bar graphic data show that appreciable numbers of IL-22-producing T cells directly measured in medium alone were comparable with those detected in medium plus CD28/CD49d mAbs or in medium plus CD3/CD28 mAbs. Data were mean ± SEM from PBMC obtained at necropsy (8 weeks after infection) from nine cynomolgus macaques. Similar data were seen at weeks 4 and 6 after *M. tuberculosis* infection. Data were gated on lymphocytes. (**C**) Representative flow-cytometry histograms of intracellular cytokine staining analysis show that IL-22- and IL-17-producing T cells appear to be two distinct cell populations. The percentage numbers of IL-22 (bottom, right), IL-17 (top, left) producing T cells and IL-22/IL-17 co-expressing T cells (top, right) in CD3 T cells are marked in the individual histograms. Data are CD3 gated. Similar data were repeatedly seen from at least 15 monkeys in blood (n = 21), BAL fluid (n = 21), lung (n = 15), spleen (n = 17), mesenteric lymph node (n = 20). (**D**) Bar graphic data show percentage numbers of T cells capable of producing IL-22 (left) and IL-17 (right) within CD3+ T cells in lymphocytes isolated from blood, BAL fluid, lungs, spleens and lymph nodes (LN) at 8 weeks after *M. tuberculosis* infection. Data are mean ± SEM derived from up to 16 macaques' lungs (n = 15, rhesus), spleens (n = 16, rhesus), mesenteric lymph nodes (n = 16, rhesus), blood (n = 9, cynomolgus), and BAL fluid (n = 9, cynomolgus). Data were measured by the ICS without antigen re-stimulation in the presence of medium plus CD28/CD49d mAb. Similar numbers of these cells were detected by the ICS in the presence of medium only (data not shown). Data were gated on CD3. *, P<0.05, **, P<0.01, ***, P<0.001. All rhesus macaques included for all the figures are Chinese rhesus macaques.

We have recently demonstrated that primary tuberculosis can induce a 220-fold up-regulation of IL-22 transcripts in macaques [14]. Another group used *in vitro* antigen stimulation-based assays to detect human T cells producing IL-22 protein in cross-sectional time points of tuberculosis [15]. Our findings at gene-expression levels suggest that T cells actively producing IL-22 *de novo* may be detected directly without *in vitro* antigen re-stimulation during *M. tuberculosis* infection of macaques. Thus, macaques were infected with *M. tuberculosis* by bronchoscope-guided inoculation as previously described [31],[32], and assessed for the development of IL-22 producing T cells using the modified ICS that skipped *in vitro* antigen re-stimulation (see Method section). Interestingly, significant increases in numbers of T cells actively producing IL-22 in blood were seen at week 4 after *M. tuberculosis* infection, and at weeks 6 and 8, such increases were more apparent and sustained in the blood of the infected macaques (Fig. 1A). Similarly, significant increases in numbers of IL-22 producing T cells were also detected in bronchoalveolar lavage fluids (BALF) during the infection (Fig. 1A). On note, these IL-22-producing T cells were similarly detected in the medium alone or medium plus CD3/CD28 mAbs (Fig. 1B, and data not shown). Most T cells actively producing IL-22 were CD4+ T cells (Fig. S1 and [3],[15]). Since it has been reported that majority IL-17 expressing T cells also co-express IL-22 [5], we similarly measured IL-17-producing T cells as described above. Surprisingly, although numbers of IL-17-producing T cells increased significantly over time after the infection, the magnitude of increase in IL-17-producing T cells was significantly lower than that in IL-22-producing T cells at weeks 6–8 after the infection (Fig. 1A). In fact, we found that most of IL-22 producing T cells were distinct from IL-17 producing T cells (Fig. 1C), with very few T cells co-expressing IL-22 and IL-17. This suggested that these IL-22 and IL-17 producing T cells may differentiate distinctly during *M. tuberculosis* infection of macaques. Thus, these results demonstrated that T cells capable of producing IL-22 or IL-17 *de novo* could be detected without *in vitro* antigen re-stimulation, and that *M. tuberculosis* infection drove significant increases in numbers of IL-22-producing T cells in the blood and airway.

### T cells actively producing IL-17 or IL-22 were preferentially increased in the lung with severe tuberculosis lesions, whereas IL-22-producing T cells were similarly distributed in the blood and lymphoid tissues

Distribution of IL-22-producing T cells in lymphoid and nonlymphoid compartments during *M. tuberculosis* infection remains largely unknown. It is not known either whether tuberculosis lesions in lung infection sites can preferentially drive expansion of T effectors cells actively producing IL-22 or IL-17. To address these questions, we isolated lymphocytes from BAL fluid, and right caudal lung lobe (infection site), spleen and mesentery lymph nodes obtained from the *M. tuberculosis*-infected macaques, and measured T cells actively producing IL-17 or IL-22 without *in vitro* antigen re-stimulation. All the macaques showed severe TB lesions in the infection site ([31],[32] and data not shown). While blood and lymphoid tissues accommodated similar numbers of T cells actively producing IL-22, lymphocytes in lungs and BAL fluid contained significantly greater numbers of IL-22-producing T cells (Fig. 1D). In fact, IL-17-producing T cells were also apparently detectable in lung lymphocytes, although numbers of these cells were quite low in the blood, spleen and mesentery lymph nodes (Fig. 1D). Overall, numbers of IL-22-producing T cells were consistently higher than those of IL-17-producing T cells in all anatomic compartments tested (Fig. 1D).

**Figure 2:**
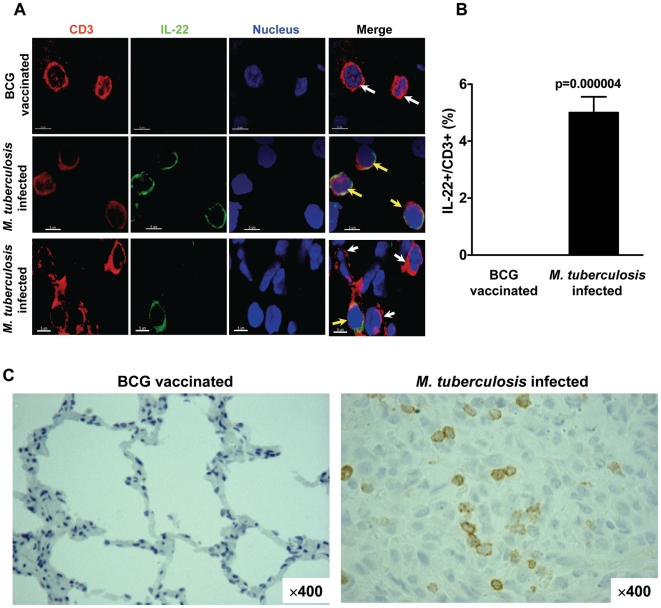
IL-22-producing T cells were detected *in situ* in lung TB granulomas from *M. tuberculosis* infected monkeys. (**A**) Representative confocal microscopic images (63× numerical aperture) at the middle and bottom panels show CD3+IL-22+ T cells (marked by yellow arrows) in lung tissue sections prepared from the right middle lung lobes at 8 weeks after *M. tuberculosis* infection in rhesus monkeys. The images at the top panel show that no IL-22 (white arrows) was detectable in CD3 T cells in the peri-bronchiolar lymphoid lung section derived from healthy macaques that received BCG vaccination 4 years before. Bar: 5 um. (**B**) Confocal imaging analyses show percentage numbers of IL-22-producing T cells in the CD3+ T cells in right middle and caudal lung lobe tissues from infected rhesus macaques and undetectable numbers of these cells in lung sections from healthy BCG-vaccinated macaques. Shown are mean percentage numbers in CD3+ T cells calculated from three *M. tuberculosis*-infected macaques and three healthy BCG vaccinated controls (see Methods). IL-22-producing T cells and IL-22 negative CD3+ T cells were counted from at least 20 confocal-section images in each of six different tissue sections from each macaque; the data from three macaques were then calculated for means, SEM, and p values. No IL-22 staining with very low background-level fluorescence was observed when using control isotype IgG and the antibody non-reactive with tissue-sectioning IL-22 (Data not shown). (**C**) Representative single-color immunohistochemistry imaging (x400) shows that IL-22-producing T cells (brown) were distributed in granuloma from the right caudle lung lobe (infection site) of one infected rhesus macaque (animal RH 7734, right). Similar staining data were seen in the lung sections from other *M. tuberculosis*-infected macaques. No IL-22-producing T cells were detectable in lung tissues from healthy BCG vaccinated rhesus macaques (representative animal RH 27097, left). RH (Chinese rhesus macaque).

To confirm the presence of T cells actively producing IL-22 in lung tissues, we performed *in situ* detection of IL-22-producing T cells using confocal microscopy imaging [33] and immunohistochemistry [31],[32]. IL-22 protein could readily be detected in CD3+ T cells in the lung tissue sections from the right middle and caudal lobes of *M. tuberculosis*-infected monkeys but not from healthy BCG-vaccinated monkeys (Fig. 2A). The mean percentage numbers of CD3+IL-22+ T cells were up to 6% in CD3+ T cells (Fig. 2B). Similarly, the immunohistochemistry studies showed that a number of IL-22-producing T cells were distributed in lung TB granuloma in the right caudal lobe from *M. tuberculosis*-infected monkeys but not in the lungs from the BCG-vaccinated controls (Fig. 2C), indicating that IL-22-producing T cells were present in TB granuloma. Taken together, T cells actively producing IL-17 or IL-22 were preferentially expanded in the lung infection site with tuberculosis lesions, whereas IL-22-producing T cells were similarly distributed in the blood and lymphoid tissues.

### Phosphoantigen HMBPP activation of Vγ2Vδ2 T cells down-regulated the capability of T cells to actively produce IL-22 but not IL-17 *de novo* in lymphocytes from blood, lung/BAL fluid, spleen and lymph nodes

**Figure 3:**
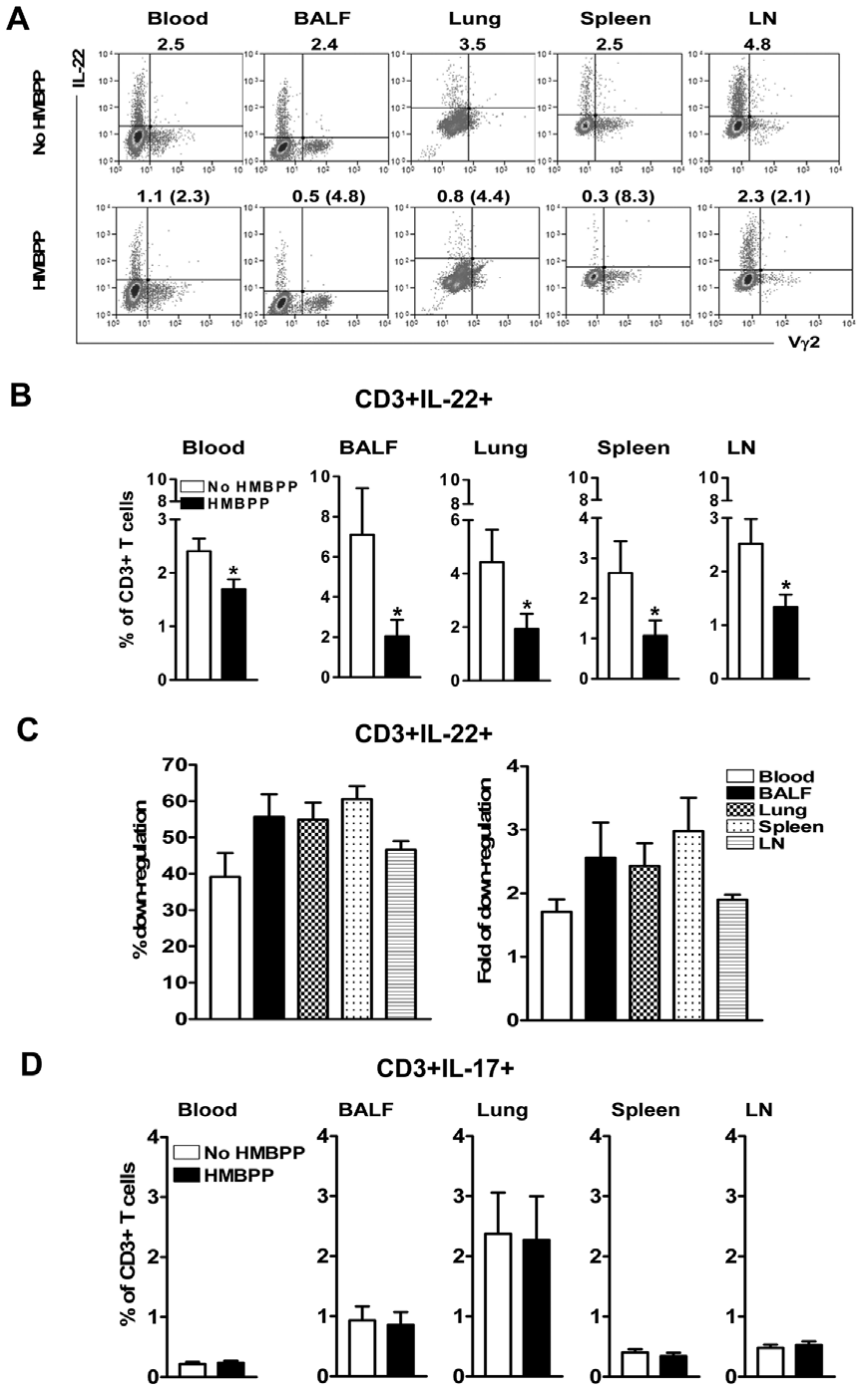
Phosphoantigen HMBPP activation of Vγ2Vδ2 T cells down-regulated the capability of T cells to actively produce IL-22 but not IL-17 *de novo* in lymphocytes from blood, lungs/BAL fluid, spleens and lymph nodes. Cells from different compartments were either un-stimulated (CD28/CD49d) or stimulated with HMBPP (HMBPP plus CD28/CD49d), and then subjected to intracellular cytokine staining. Blood and BALF were taken at the endpoint matching necropsy schedule after *M. tuberculosis* infection. (**A**) Shown on the top panel are representative flow-cytometry histograms of intracellular cytokine staining analysis. The percentage numbers of IL-22-producing T cells in lymphocytes are marked above individual histograms. The bottom-panel flow-cytometry histograms show that HMBPP activation resulted in a reduction in numbers of T cells capable of producing IL-22. The numbers in parentheses denote folds of reduction (ratio of numbers of IL-22-producing T cells from unstimulated versus HMBPP stimulated measurements) after HMBPP treatment. Data were gated on CD3. The flow histograms were from representative *M. tuberculosis*-infected macaque CN7234 (blood), CN7222 (BAL fluid), RH7716 (lung), RH7717 (spleen) and RH7720 (mesenteric LN) respectively. CN (cynomolgus macaque). (**B**) Comparative analyses of IL-22-producing T cells in the presence and absence of HMBPP stimulation. Data are mean percentage numbers ± SEM derived from macaque animals' blood (n = 7, cynomolgus), BAL fluids (n = 5, cynomolgus), lungs (n = 7, rhesus), spleens (n = 11, rhesus) and mesenteric lymph nodes (n = 8, rhesus). *, P<0.05, **, P<0.01. Data were gated on CD3. (**C**) Bar graphic data show percentages (left) and folds (right) of down-regulation of IL-22-producing T cells after HMBPP stimulation. The fold of down-regulation was defined the same as above in Fig. 3A. The percentages of down-regulation were calculated by the formula [(Unstimulated–HMBPP-stimulated)/Unstimulated × 100%]. Results were shown as mean ± SEM. Data were gated on CD3. (**D**) Comparative analyses of IL-17-producing T cells in the presence and absence of HMBPP stimulation. Data are mean percentage numbers ± SEM derived from macaque animals' blood (n = 9, cynomolgus), BAL fluids (n = 9, cynomolgus), lungs (n = 15, rhesus), spleens (n = 16, rhesus) and mesenteric lymph nodes (n = 20, rhesus). Data were gated on CD3.

**Figure 4 ppat-1000789-g004:**
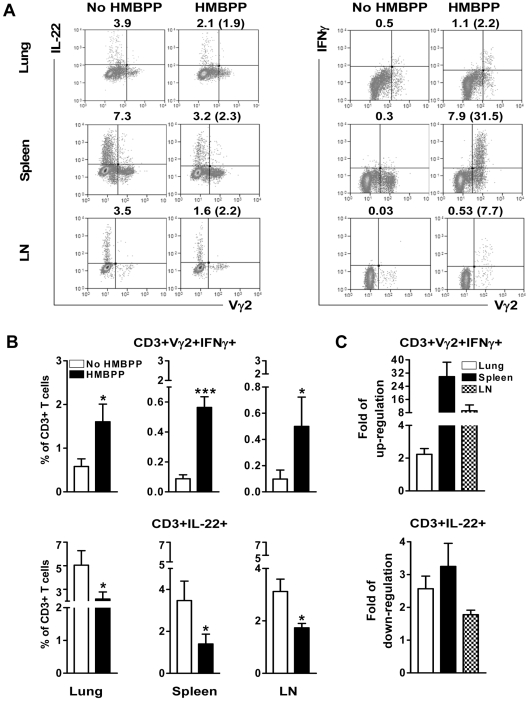
Up-regulation of IFNγ-producing Vγ2Vδ2 T effector cells after HMBPP stimulation coincided with the down-regulated capacity of T cells to produce IL-22 *de novo*. (**A**) Representative paired flow-cytometry histograms from monkey RH7406 (lung), RH7720 (spleen), RH7357 (mesenteric lymph node) show intracellular cytokine staining data indicating that HMBPP stimulation up-regulated numbers of CD3+ IFNγ+Vγ2+ T cells (right) and down-regulated numbers of CD3+IL-22+ T cells (left) in lymphocytes from lungs, spleens and mesenteric lymph nodes (LN). Cells were gated on CD3. Percentage numbers of IL-22+ or IFNγ+Vγ2+ T cells in CD3 T cells are listed above individual histograms, and folds of up-regulation (the ratio of HMBPP-stimulated versus unstimulated) or down-regulation are indicated in parentheses. Note that 1 hour HMBPP stimulation did not increase the number of entire Vγ2Vδ2+ T cells (including both IFNγ+ and IFNγ- Vγ2Vδ2+ T-cell populations) in CD3+; the percentage of Vγ2Vδ2+ T cells in CD3+ remained similar in the presence or absence of HMBPP (HMBPP enabled some IFNγ- Vγ2Vδ2+ T cells to become IFNγ+ Vγ2Vδ2+ T cells). The decreased numbers of IL-22-producing T cells were not due to expansion of Vγ2Vδ2+ T cells after 1-hr HMBPP activation. (**B**) Comparative analyses of flow-cytometry data indicating that HMBPP stimulation up-regulated percentage numbers of IFNγ+Vγ2+ T cells (top panels) and down-regulated percentage numbers of IL-22+ CD3+ T cells (bottom) in lymphocytes from lungs (n = 6), spleens (n = 8) and mesenteric lymph nodes (n = 5). Data were means ± SEM derived from eight infected rhesus macaques and were gated on CD3. *, P<0.05, ***, P<0.001. (**C**) Comparative data indicating mean folds of up-regulation of CD3+IFNγ+Vγ2+ T cells (top) and down-regulation of CD3+IL-22+ T cells in lymphocytes from lungs (n = 6), spleens (n = 8) and mesenteric lymph nodes (n = 5). Data were derived from eight infected rhesus macaques and were gated on CD3.

**Figure 5:**
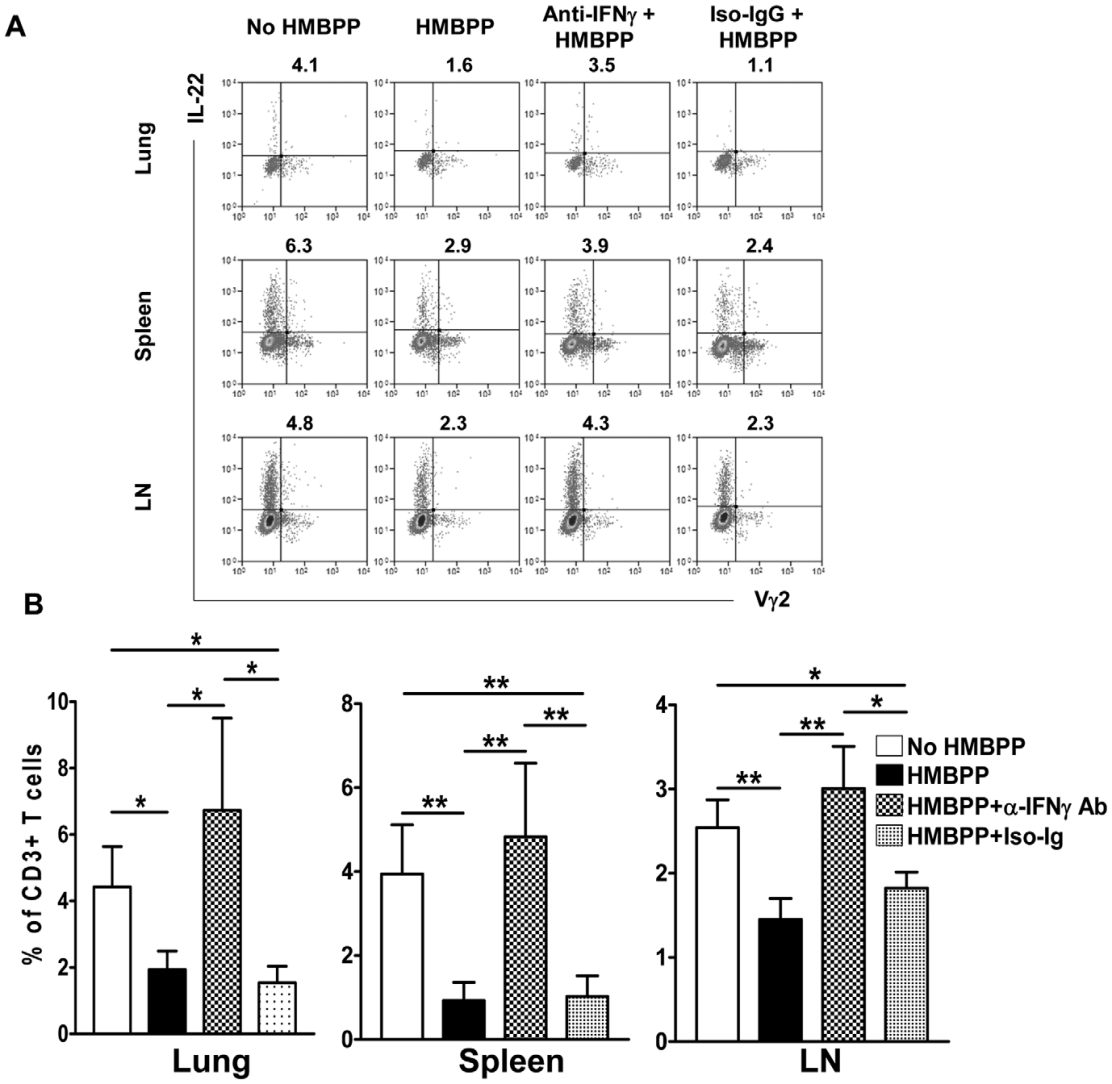
Anti-IFNγ neutralizing Ab treatment reversed the HMBPP-mediated down-regulation effect on IL-22-producing T cells. Cells from different anatomic compartments were either un-stimulated or stimulated with HMBPP, HMBPP + anti-IFNγ neutralizing Ab, or HMBPP + isotype matched IgG Ab, and then subjected to intracellular cytokine staining. (**A**) Representative flow-cytometry histograms from monkey RH7717 (lung), RH7719 (spleen) and RH7720 (mesenteric lymph node) show intracellular cytokine staining data demonstrating that anti-IFNγ neutralizing Ab but not isotype IgG reversed the HMBPP-mediated down-regulation of IL-22+CD3+ T cells in lymphocytes from lungs, spleens and mesenteric lymph nodes (LN). Cells were gated on CD3. Percentage numbers of CD3 T cells are listed above individual histograms. (**B**) Comparative flow-cytometry data show that anti-IFNγ Ab but not isotype IgG reversed the HMBPP-mediated down-regulation of IL-22+CD3+ T cells in lymphocytes from lungs, spleens and mesenteric lymph nodes (LN). Data were mean ± SEM derived from rhesus macaque animals' lungs (n = 7), spleens (n = 9) and mesenteric lymph nodes (n = 14). *, P<0.05, **, P<0.01. Addition of exogenous IFNγ to PBMC culture was not able to activate/expand Vγ2Vδ2 T cells or to down-regulate mature IL-22-producing T cells (data not shown).

The immune regulatory factors regulating T cells actively producing IL-22 during infections have not been studied. Because Vγ2Vδ2 T cells recognize phosphoantigen HMBPP produced by *M. tuberculosis* and other microbes, and contribute to adaptive immune responses and immune regulation in mycobacterial infections [21],[30], we sought to determine whether phophoantigen activation of Vγ2Vδ2 T cells can exert a potential impact on IL-22-producing T cells. Our finding that IL-22-producing T cells and Vγ2Vδ2 T cells are both distributed in lungs, blood and lymphoid tissues (Fig. 3A, 4A, 5A and [31]) in *M. tuberculosis* infection provides a useful setting in which to study the interaction between these two T-cell subpopulations. Thus, lymphocytes isolated from the blood, lung/BAL fluid, spleen and lymph nodes of the infected macaques were stimulated for one hr with HMBPP, and then assessed for a change in the capability of T cells to produce IL-22 *de novo* in comparison with that of lymphocytes without Ag stimulation. Interestingly, HMBPP activation of Vγ2Vδ2 T cells consistently led to down-regulation of the capability of T cells to produce IL-22 *de novo* in lymphocytes from blood, lung/BAL fluid, spleen and mesenteric lymph nodes (Fig. 3A, 3B). The percentage numbers of T cells actively producing IL-22 were reduced 1.4–7.9 folds after HMBPP stimulation, with mean reduced folds being 1.71±0.20 in blood (p = 0.0166), 2.56±0.55 in BAL fluid (p = 0.0249), 2.43±0.36 in lung (p = 0.0430), 2.98±0.53 in spleen (p = 0.0446), and 1.90±0.08 in lymph nodes (p = 0.0188) when compared to corresponding cell numbers detected without HMBPP stimulation (Fig. 3A, 3B and 3c). In contrast, HMBPP activation of Vγ2Vδ2 T cells had almost no impact on the capacity of T cells to produce IL-17 from the blood, lung/BAL fluid, spleen and lymph node (Fig. 3D). This result suggested that HMBPP activation of Vγ2Vδ2 T cells selectively down-regulated IL-22-producing T cells but not IL-17-producing T cells. Overall, the HMBPP-mediated reduction in *de novo* production of IL-22 by T cells was seen in lymphocytes from all the compartments, but the down-regulation appeared more dramatic in lymphocytes from lung/BAL fluid and spleen (Fig. 3A, 3B). This might be due to the fact that greater numbers of Vγ2Vδ2 T cells were present in these compartments in tuberculosis [31]. Stimulation of lymphocytes using overlapping ESAT6/Ag85 peptides did not lead to significant reduction in IL-22-producing T cells (data not shown). These results therefore demonstrated that HMBPP activation of Vγ2Vδ2 T cells down-regulated the capability of T cells to actively produce IL-22 *de novo* during *M. tuberculosis* infection.

### Up-regulation of IFNγ-producing Vγ2Vδ2 T effector cells after HMBPP stimulation coincided with the down-regulated capacity of T cells to produce IL-22 *de novo*

We then asked the question as to whether effector function of Vγ2Vδ2 T cells after HMBPP stimulation contributed to the down-regulated capability of T cells to actively produce IL-22 *de novo*. Since IFNγ is one of the major cytokines produced by phosphoantigen-activated Vγ2Vδ2 T cells [16],[29], we sought to determine whether there was a connection between the increase in IFNγ-producing Vγ2Vδ2 T effector cells and the down-regulation of IL-22-producing-T cells after HMBPP stimulation. To this aim, we measured the numbers of IL-22-producing T cells and IFNγ-producing Vγ2Vδ2 T effector cells after HMBPP stimulation of lymphocytes from lung, spleen and mesenteric lymph nodes in comparison with those without HMBPP stimulation. Interestingly, while the numbers of T cells producing IL-22 dropped significantly after HMBPP stimulation of lymphocytes, with mean reduced folds being 2.57±0.39 in lungs (p = 0.0316), 3.25±0.71 in spleens (p = 0.0331) and 1.78±0.14 in mesenteric lymph nodes (p = 0.0126), the numbers of IFNγ-producing Vγ2Vδ2 T cells increased dramatically in all these HMBPP-stimulated lymphocyte populations (Fig. 4A, 4B and 4C). The increase in numbers of IFNγ-producing Vγ2Vδ2 T effector cells was most significant in spleen lymphocytes (29.83±8.71-fold increase, p<0.0001) when compared with the numbers without HMBPP stimulation (Fig. 4C). Two- to ten-fold increases in IFNγ-producing Vγ2Vδ2 T cells were also seen after HMBPP-stimulation of lymphocytes from lungs (2.23±0.35, p = 0.0217) and lymph nodes (9.17±3.55, p = 0.0311) (Fig. 4C). We also measured production of cytotoxic molecule perforin after HMBPP stimulation to examine if cytotoxic effector of Vγ2Vδ2 T cells contributed to the down-regulation of IL-22-producing T cells. No significant changes in numbers of perforin-producing Vγ2Vδ2 T cells from blood and BAL fluid were seen after HMBPP stimulation (data not shown). These data provided evidence demonstrating inverse relationship between IFNγ-producing Vγ2Vδ2 T effector cells and IL-22-producing T cells, suggesting that effector function of Vγ2Vδ2 T cells after HMBPP stimulation contributed to the down-regulation of the capability of T cells to actively produce IL-22 *de novo*.

### Anti-IFNγ neutralizing Ab treatment reversed the HMBPP-mediated down-regulation effect on IL-22-producing T cells

Given the possibility that effector function of Vγ2Vδ2 T cells after HMBPP activation contributed to the down-regulation effect on IL-22-producing T cells, the central question now was whether IFNγ networking function or cytotoxic activity of Vγ2Vδ2 T cells mediated the down-regulation of IL-22-producing T cells. Since HMBPP-activated Vγ2Vδ2 T cells from mycobacterium-infected macaques do not exhibit cytotoxic activity against autologous CD4 T cells [30] (data not shown), we focused on the mechanistic studies determining whether blocking the effector function of IFNγ production by Vγ2Vδ2 T cells could reverse the down-regulation effect on IL-22-producing T cells. We therefore performed cytokine neutralization experiments using anti-IFNγ neutralizing antibody [30], and examined if neutralization of IFNγ could abrogate HMBPP-mediated down-regulation of IL-22-producing T cells. Interestingly, anti-IFNγ neutralizing antibody effectively reversed the HMBPP-mediated down-regulation of IL-22-producing T cells (Fig. 5A, 5B). In fact, the blocking effect exerted by anti-IFNγ neutralizing antibody was so dramatic that the numbers of T cells capable of producing IL-22 in the lymphocytes after the HMBPP + anti-IFNγ Ab co-treatment were comparable to those seen without HMBPP stimulation in lymphocytes from lungs, spleens, and mesenteric lymph nodes (Fig. 5A, 5B). In contrast, control isotype antibody had no detectable effect on IL-22-producing T cells (Fig. 5A, 5B). These results suggested that γδ T-cell-driven IFNγ-networking function was one of the mechanisms by which phosphoantigen-activated Vγ2Vδ2 T effector cells down-regulated the capability of T cells to produce IL-22 *de novo* during *M. tuberculosis* infection.

## Discussion

The current study represents the first extensive investigation of the development, distribution, and immune regulation of IL-22-producing T cells differentiated from primary *M. tuberculosis* infection. Interestingly, tuberculosis-driven T cells can actively produce IL-22 or IL-17 *de novo* without the requirement of *in vitro* antigen re-stimulation. The increase in numbers of IL-22 or IL-17 producing effector T cells is particularly evident in the lung tissues during the late stage of *M. tuberculosis* infection. More importantly, *in situ* studies using confocal microscopy and immunohistochemistry can readily detect these IL-22-producing T cells in lung tissue sections and granulomas (Fig. 2A, 2B, and 2C) from infected monkeys, indicating that IL-22-producing T cells are present in TB granulomas or lesions. These findings are quite contrasted to the recent report showing that Th17 cells in *M. tuberculosis*-infected individuals are detected under the condition of *in vitro* antigen re-stimulation [15]. It is generally believed that cytokine proteins produced by CD4 T cells usually can not be detected directly *in vivo* unless they receive potent *in vitro* Ag re-stimulation. The presence of large numbers of IL-22-producing T cells in lungs suggests that *M. tuberculosis* antigens may have activated these cells to a great extent so that they are capable to produce large quantity of IL-22 or IL-17 *in vivo*. This notion is consistent with greater numbers of IL-22- or IL-17-producing T cells distributed in lung tissues with high TB burden and severe TB lesions. These T cells appear to be fully matured or differentiated after exposure to *M. tuberculosis*, as *in vitro* stimulation by triggering TCR/CD3 signaling using anti-CD3/CD28 do not dramatically increase or decrease the number of IL-22-producing T cells in lymphocytes (Fig. 1B, and data not shown).

It is noteworthy that human IL-22-producing Th17 cells detected at cross-sectional time points in patients with active TB are lower than those in healthy mycobacterium-exposed individuals [15]. The explanation for this may be simply due to the difference in immune competence between chronically-active adult tuberculosis in humans and primary *M. tuberculosis* infection in macaques. Chronically active adult TB usually occurs as a result of reactivation TB somehow after immune control of primary *M. tuberculosis* infection, and often display depressed cellular immune response [34],[35]. Therefore it is not surprised that the levels of IL-22 and IL-17 producing Th17 cells in active TB patients are lower than those in *M. tuberculosis*-exposed healthy controls. Tuberculosis-driven IL-10-producing T cells might down-regulate human IL-22 and IL-17 producing Th17 cells in chronically-active TB [36]. In contrast, the macaques in our studies were naïve at the time of experimental *M. tuberculosis* infection, and were able to develop competent immune responses, characterized by the increased numbers of IFNγ+ and TNFα+ Th1 cells in the blood and lungs following *M. tuberculosis* infection (data not shown). Furthermore, IL-10-producing T cells in this study are almost undetectable or extremely low during primary *M. tuberculosis* infection (data not shown). Therefore, these differences may help to explain why we see apparent increases in IL-22-producing T cells during primary *M. tuberculosis* infection of naïve macaques, whereas chronically-active TB patients display depressed responses of IL-22-producing T cells.

Our study provided the first evidence that T cells actively producing IL-22 are susceptible to down-regulation by immune activation of certain T effector subsets. We have shown that the capability of T cells to produce IL-22 can be down-regulated by phosphoantigen activation of Vγ2Vδ2 T effector cells. Such relationship between IL-22-producing T cells and HMBPP activation of Vγ2Vδ2 T effector cells may somehow predict the *in vivo* cell-cell interaction in lung and lymphoid tissues. In fact, these two different T effector cell subpopulations co-exist in all anatomic compartments tested during *M. tuberculosis* infection (Figs. 3–5). Particularly, these two T cell subpopulations producing their own effector cytokines can be detected *in situ* in *M. tuberculosis* infected lung tissues and TB granulomas (Fig. 2A, 2C, [31]). The negative effects of Vγ2Vδ2 T effector cells on IL-22-producing T cells underscores broad regulatory function of Vγ2Vδ2 T effector cells as phosphoantigen-activation of Vγ2Vδ2 T cells can also antagonize IL-2-induced CD4+CD25+Foxp3+ T regulatory cells in mycobacterial infection [30]. On the contrary, HMBPP activation of Vγ2Vδ2 T cells did not significantly down-regulate IL-17-producing T cells. While the mechanism for the selective down-regulation of IL-22-producing T cells is unknown, it is possible that IL-22-producing T cells and IL-17-producing T cells are two distinct T cell subsets in macaques, and therefore regulated by different mechanisms [15]. This is consistent with the report describing distinct IL-22 and IL-17 producing Th17 cells among CD4 T cells in active pulmonary TB patients [15]. It was noteworthy that in order to perform consistent or comparable evaluation of T effector cells in different compartments in TB, we collected blood and BALF (Fig. 3) at the end point matching the necropsy schedule to study the interaction between Vγ2Vδ2 T cells and IL-22/IL-17-producing T cells. The frequency of IL-22-producing T cells at the end stage might be highest (Fig. 1A) in all different compartments so that γδ T-cell-driven down-regulation of IL-22-producing T cells was quite evident.

Our mechanistic study in IFNγ-neutralizing experiments demonstrate that IFNγ networking function from HMBPP-activated Vγ2Vδ2 T effector cells can effectively down-regulate the IL-22 production effector function of IL-22-producing T cells that are matured or differentiated during *M. tuberculosis* infection. Molecular pathways for IFNγ networking function from Vγ2Vδ2 T-cells in down-regulation of IL-22 producing T cells may be complex, since exogenous IFNγ is not able to activate/expand Vγ2Vδ2 T cells or to down-regulate mature IL-22-producing T cells (data not shown). We cannot exclude the possibility that endogenous IFNγ acts in concert with other cytokines produced by Vγ2Vδ2 T effectors to down-regulate IL-22-producing T cells. The current finding suggests that IFNγ networking function from HMBPP-activated Vγ2Vδ2 T cells serves at least as a negative host factor for down-regulation of IL-22 producing T cells during *M. tuberculosis* infection. The γδ T-cell-driven IFNγ networking function may be significant for the potential *in vivo* regulation of IL-22-producing T cells by Vγ2Vδ2 T effector cells. HMBPP produced by *M. tuberculosis* metabolite pathway can activate Vγ2Vδ2 T cells with endogenous production of IFNγ and other effectors, leading to down-regulation of IL-22-producing T cells.

Given the possibility that IL-22-producing T cells may be either inflammatory/pathogenic or protective during infections depending upon bacterial burdens and host conditions, HMBPP-activated Vγ2Vδ2 T effector cells may play a regulatory role in balancing IL-22-mediated inflammatory and anti-microbial responses. During a *M. tuberculosis* infection with low-level bacterial burdens, activation extent of Vγ2Vδ2 T cells and IL-22-producing T cells would be low or limited due to low-level production of HMBPP and protein Ags. Such low-degree activation of Vγ2Vδ2 T cells would lead to no or subtle down-regulation of IL-22 production. Thus, low or moderate levels of IL-22 may facilitate potential innate responses by promoting pulmonary epithelial cells' capacity to produce antimicrobial peptides [3],[6],[7],[37]. On the other hand, high bacterial burdens during severe *M. tuberculosis* infection may induce unbalanced overproduction of IL-22 and severe lesions [14], an outcome consistent with IL-22-mediated inflammatory responses and diseases [8],[9],[37]. In this case, potent activation of Vγ2Vδ2 T cells driven by high-level production of HMBPP in severe tuberculosis may potentially down-regulate IL-22-production and antagonize IL-22-producing T cell-mediated inflammation. From this standpoint, the findings from the current work raise the possibility to determine whether the HMBPP treatment regimen for expanding Vγ2Vδ2 T effector cells [29] may confer some therapeutic benefits regulating tuberculosis lesions.

Thus, we have demonstrated differentiation, local/systemic distribution and immune regulation of mature T cells capable of producing IL-22 *de novo* during *M. tuberculosis* infection. Particularly, we have illustrated that phosphoantigen activation of Vγ2Vδ2 T cells can down-regulate IL-22-producing T cells through IFNγ-networking function. Since the role of IL-22-producing T cells in *M. tuberculosis* infection remains unknown in humans, our current findings provide a useful system to further understand the interplay between IL-22-producing T cells and Vγ2Vδ2 T effector cells in *M. tuberculosis* infection and their mutual contribution to anti-tuberculosis responses.

## Materials and Methods

### Ethics statement

The use of macaques and experimental procedures were approved by Institutional Animal Care and Use Committee and Biosafety Committee, University of Illinois College of Medicine at Chicago (UIC), and we followed the national and international guidelines regarding “The use of non-human primates in research to minimize potential suffering of the studied macaques. Daily or weekly clinical follow-up were taken to ensure that animals were not suffering from severe coughing, respiratory distress, depression, refusion to take food, body-weight loss or other potential life-threatening signs. Human euthanization procedures were immediately taken if those signs occur progressively.

### Animals

Fifteen adult cynomolgus macaques ranging in age from 3 to 9 years old and eighteen adult Chinese rhesus macaques monkeys ranging in age from 4 to 11 years old were used in this study. Cynomolgus and rhesus macaques exhibited similar responses of IL-22-producing T cells and Vγ2Vδ2 T cells after *M. tuberculosis* infection. All monkeys were naïve prior to *M. tuberculosis* infection, based on tuberculin skin tests, IFNγ ELISOPOT assays, and thoracic radiographs. *M. tuberculosis* infected macaques were housed at the Biologic Research Resources Annex BSL3 nonhuman primate facilities in UIC and sacrificed at 8 weeks after infection. As a control, tissues sections for *in situ* IL-22 detection were prepared from lung tissues of 3 healthy rhesus monkeys, which were vaccinated intravenously with 10^6^ CFU BCG Pasteur 4 years earlier (Fig. 2). As another control, BAL fluid was collected from nine naïve cynomolgus macaques to measure IL-17- or IL-22-producing T cells (Fig. 1A).

### *M. tuberculosis* infection

Each monkey was infected with 500 CFU of *M. tuberculosis* Erdman (the standard challenge stock from FDA) by the bronchoscope-guided injection of the inoculum into the right caudal lobe as previously described [31],[32]. This was done in the procedure room at the Annex BSL3 nonhuman primate facilities at UIC. The inoculum used for infection was diluted and plated on 7H11 agar plates (BD) to further confirm the bacterial CFU dose for inoculation.

### Bronchoalveolar lavage (BAL)

This was done essentially the same as previously described [29],[31].

### Isolation of lymphocytes from blood, BAL fluid, lung, spleen and mesenteric lymph nodes

PBMC were isolated from freshly collected EDTA blood by Ficoll-Paque ^plus^ (Amersham, Piscataway, NJ) density gradient centrifugation. For isolation of lymphocytes from BAL fluid, freshly retrieved BAL fluid was filtered through 40-um cell strainers (BD) into 50 ml-conical tubes (BD) followed by 5 min ×1500 rpm centrifugation. The supernatant was gently discarded without disturbing the cell pellets. Cell pellets were then treated with 5 ml RBC blood lysis buffer (Sigma-Aldrich) for 10 min or waited till the suspension became clear and washed once with 5% FBS-PBS. Lung and spleen tissues were minced with sharp scissors and squeezed with sterile copper mesh in Petri dish and colleted with RPMI followed by filtering through 40-um cell strainers. Mesenteric lymph nodes were carefully teased apart with 18-gauge needle in Petri dish with RPMI to form single cell suspension. Collected cell suspensions from lung, spleen and mesenteric lymph nodes were further purified by Ficoll-Paque ^plus^ density gradient centrifugation to collect pure lymphocytes. The freshly isolated lymphocytes from blood, BAL fluid, lung, spleen and mesenteric lymph nodes were stained by trypan blue to identify the viability and numerate cell counts. The lymphocytes were finally suspended into 10% FCS-RPMI media with a concentration of 10^7^ cells/ml for further study.

### Antigens (Ag) and Antibodies (Ab)

Phosphoantigen compound HMBPP was produced, characterized, validated and provided by Dr. Hassan Jomaa from Justus-Liebig-Universität Giessen, Giessen, Germany. HMBPP used in the study was >98% pure [29], and specifically stimulated activation/expansion of Vγ2Vδ2 T cells but not other cell subpopulations[28]. This allowed us to determine if HMBPP activation of Vγ2Vδ2 T cells in lymphocytes could down-regulate IL-22-producing T cells in the CD3+ T-cell pool. PPD and 15mer overlapping peptides spanning Ag85B were purchased from Mycos Research (Loveland, CO) and GenScript Corporation (NJ), respectively. Anti-CD28 (CD28.2, BD) and anti CD49d (9F10, BD) were used in the assays as costimulatory Abs. The following Abs were used for surface and intracellular cytokine staining for flow cytometry: CD3-PECY7 (SP34-2, BD), CD4-FITC (L200, BD), Vγ2-FITC (7A5, Endogen), IFNγ-Allophycocyanin (4S.B3, BD), IL-17-PE (eBio64CAP17, eBioscience), IL-22-biotinylated (anti-human, RD), streptavidin-Pacific blue (invitrogen). Anti-IFNγ neutralizing Ab (MD-1, eBioscience) and purified mouse IgG isotype control (eBioscience) were used in blocking assay.

### Intracellular cytokine staining (ICS) assays, anti-IFNγ neutralizing Ab blocking assay, and control experiments

10^6^ lymphocytes isolated from blood, 10^6^ or 3–5×10^5^ lymphocytes (depending on availability) from BAL fluid, lung, spleen and mesenteric lymph node were used in each reaction (round bottom 96-well plate) to measure IL-22- or IL-17-producing T cells as well as IFNγ- or perforin-producing Vγ2Vδ2 T cells. Lymphocytes were incubated for one hr with (i) medium alone (termed No CD28/CD49d), (ii) medium plus co-stimulatory CD28 (1 ug/ml) and CD49d (1 ug/ml) mAbs (termed CD28/CD49d), or (iii) HMBPP (40 ng/ml) plus CD28/CD49d mAbs (termed HMBPP). For confirmation purposes, lymphocytes were also incubated for one hr with medium plus CD3 (1 ug/ml) and CD28 (1 ug/ml) mAbs (termed CD3/CD28), PPD (20 ug/ml) plus CD28/CD49d mAbs, or 15mer overlapping peptides spanning Ag85B (2 ug for each peptide) plus CD28/CD49d mAbs. The initial one-hour incubation was carried out in a 200 ul final volume in round bottom 96-well plates at 37°C, 5% CO_2_, followed by five hr incubation in the presence of brefeldin A (GolgiPlug, BD). We found that at late time points after *M. tuberculosis* infection, appreciable numbers of IL-22-producing T cells could be directly measured by the intracellular cytokine staining (ICS) without *in vitro* antigen stimulation, and that numbers of IL-22-producing T cells directly measured in medium only were comparable with those detected in medium plus CD28/CD49d mAbs or medium plus CD3/CD28 mAbs (Fig. 1B, and data not shown).

For anti-IFNγ neutralizing antibody blocking assay, 10^6^ or 3–5×10^5^ lymphocytes from lung, spleen and mesenteric lymph node were treated as a test group with anti-IFNγ neutralizing Ab (5 ug/ml) together with HMBPP (40 ng/ml) in presence of CD28/CD49d mAbs. As control groups, cells were treated with medium plus CD28/CD49d mAbs only, HMBPP plus CD28/CD49d mAbs, or HMBPP plus mouse isotype IgG (5 ug/ml) and CD28/CD49d mAbs. The 1 hr reaction was followed by 5 h incubation with brefeldin A as described above.

After a total of six hr incubation, cells in 96-well plate were transferred into 5 ml polystyrene round bottom tubes (BD) for surface and intracellular staining. Cells were washed once with 2% FBS-PBS and stained at RT for at least 15 min with surface marker Abs (CD3, CD4 and Vγ2) followed by twice wash with 2% FBS-PBS. Cells were permeabilized for 45 min (cytofix/cytoperm, BD) and washed twice by Perm buffer (BD), and then stained another 45 min for IFNγ, IL-17 and IL-22-biotinylated and repeated Perm wash twice. Cells stained with biotinylated-IL-22 were further stained for 45 min with streptavidin-pacific blue conjugate followed by final twice Perm buffer wash. Last, cells were resuspended in 2% formaldehyde-PBS (Protocol Formalin, Kalamazoo, MI) and subjected to flow cytometry analysis.

To ensure the specific immune staining in ICS, matched normal serum or isotype IgG served as negative controls for staining cytokines or surface markers. As *in vivo* control experiments, PBMC were obtained biweekly for 8 weeks from three healthy uninfected and five SHIV-infected cynomolgus macaques, and assessed for IL-22-producing T cells using the ICS assay as described above (medium only or medium plus CD28/CD49d). No or very few IL-22-producing T cells (<0.02% of CD3) were detectable in the longitudinal control experiments (data not shown).

### Flow cytometry analysis

Fixed lymphocytes were run on a CyAn ADP flow cytometer (DakoCytomation, Carpinteria, CA) for analysis. Lymphocytes were gated based on their forward scatter, side scatter and pulse-width characteristics. At least 40,000 gated events were analyzed using Summit Data Acquisition and Analysis Software created by DakoCytomation. Cells stained with different color conjugated Abs alone were used as controls and to estimate the amount of compensation needed for the different color combinations. Further gating and determination of quadrant position for analysis were based on specific Ab staining (positives) together with negative background determined by either unstained cells or isotype control stained cells. Flow cytometric dot plots were displayed by bi-exponential scaling. Since IL-22-producing T cells and Vγ2Vδ2 T cells both expressed CD3, flow-cytometry gating on CD3 objectively revealed HMBPP-stimulated increases in percentages of IFNγ-producing Vγ2Vδ2 T cells and associated decreases in percentages of IL-22-producing T cells within the CD3+ T-cell population. Such interrelation between Vγ2Vδ2 T cells and IL-22-producing T cells could not be precisely determined by presenting IL-22-producing cells in CD4+ T cells because Vγ2Vδ2 T cells usually do not express CD4. Furthermore, because IL-22 was also produced by CD8 T cells and γδ T cells (Fig. S1), determining IL-22 production in CD3+ T cells would be more conclusive than in CD4+ T cells.

### Confocal microscopy imaging

≈5-µm-thick frozen lung sections were prepared, as we recently described [31], from optimal cutting temperature compound (OCT)-embedded lung tissues from healthy macaques vaccinated with BCG four year before, or *M. tuberculosis*-infected macaques. Tissue sections were first incubated overnight in a wet box with polyclonal rabbit anti-human IL-22 (N-terminus, Capralogics,) and monoclonal mouse anti-human CD3 (F7.2.38, Dako) or isotype control IgG or normal rabbit serum. After thoroughly washed with PBS, tissue sections were fixed with 2% formalin and washed thoroughly with PBS. Tissue sections were then incubated with FITC-conjugated donkey anti-rabbit IgG (Biolegend) and Cy3-conjugated goat anti-mouse IgG (Biolegend, USA), followed by thorough PBS washing. Next, tissue sections were fixed gently with 2% formalin again and washed thoroughly with dd water to remove the salt in PBS. Finally, tissue sections were mounted on slides using fluorescence mounting medium with DAPI for confocal microscope [Zeiss, LSM 510, (63× numerical aperture)] imaging.

### Immunohistochemistry analysis of IL-22 producing T cells in lung tissues

Standard protocols for immunohistochemical analyses were used to evaluate IL-22-producing T cells in lung tissue sections prepared from OCT-embedded tissues as we previously described [31]. A peroxidase-based visualization kit (Envision system K1395; Dako, Caprinteria, CA) was used for immunohistochemical staining. Frozen specimens embedded in OCT were cut into 6-um thick sections by use of a cryostat, fixed, permeabilized in cold acetone for 10 min, and washed in PBS. The sections were treated for 10 min with 1% hydrogen peroxide in PBS to quench endogenous peroxidase, rinsed in PBS, blocked for 10 min with serum-free protein block (X0909; Dako), and rinsed in PBS. The sections were incubated with rabbit anti-human IL-22 (CI0144; Capralogics) Ab at a concentration of 50 ug/ML for 1 h at room temperature and then incubated for 30 min with peroxidase-labeled polymer-conjugated goat anti-rabbit immunoglobulin. The sections were rinsed in PBS after each incubation, developed using 3,3′-diaminobenzidine chromogen solution as a substrate for 3–6 min, and counterstained with Gill's hematoxylin (Fisher Scientific) for 2 seconds. After dehydration in grade alcohols, sections were cleared in xylene and coverslipped.

### Statistical analysis

Statistical analysis was done by using GraphPad Prism software (GraphPad Software, Inc., La Jolla, CA). Normality test was first performed to decide whether the data were normally distributed. All the data in this study passed the normality test, and analyzed by Student t test (parametric method). All the P values shown in this study were derived from Student t test. P<0.05 was considered significant. Only P values <0.05 were shown in the text.

## Supporting Information

Figure S1 T cells producing IL-22 are mainly produced by CD4+ T cells during *M. tuberculosis* infection. Bar graphic data show the percentages of CD4, CD8 and Vγ2Vδ2 T cells among CD3+IL22+ T cells isolated from the mesenteric lymph nodes (n = 4, rhesus) at necropsy. Data were mean±SEM and gated on CD3.
(0.02 MB DOC)
