# Supplementary material for: Differentiation, Distribution and γδ T Cell-Driven Regulation of IL-22-Producing T Cells in Tuberculosis
Source: PLoS Pathog. 2010 Feb 26;6(2):e1000789. doi: 10.1371/journal.ppat.1000789 (PMC2829073; doi:10.1371/journal.ppat.1000789)
Supplement: Figure S1 — T cells producing IL-22 are mainly produced by CD4+ T cells during M. tuberculosis infection. Bar graphic data show the percentages of CD4, CD8 and Vγ2Vδ2 T cells among CD3+IL22+ T cells isolated from the mesenteric lymph nodes (n = 4, rhesus) at necropsy. Data were mean±SEM and gated on CD3. (0.02 MB DOC) [file ppat.1000789.s001.doc]

Supplemental Figure 1

T cells producing IL-22 are mainly produced by CD4+ T cells during *M. tuberculosis* infection. Bar graphic data show the percentages of CD4, CD8 and Vγ2Vδ2 T cells among CD3+IL22+ T cells isolated from the mesenteric lymph nodes (n=4, rhesus) at necropsy. Data were mean±SEM and gated on CD3.
